# The Influence of Zn Substitution on Physical Properties of CoFe_2_O_4_ Nanoparticles

**DOI:** 10.3390/nano13010189

**Published:** 2022-12-31

**Authors:** Adam Szatmari, Rares Bortnic, Gabriela Souca, Razvan Hirian, Lucian Barbu-Tudoran, Fran Nekvapil, Cristian Iacovita, Emil Burzo, Roxana Dudric, Romulus Tetean

**Affiliations:** 1 Faculty of Physics, “Babes Bolyai” University, Kogalniceanu 1, 400084 Cluj-Napoca, Romania; 2 Electron Microscopy Center “Prof. C. Craciun”, Faculty of Biology & Geology, “Babes-Bolyai” University, 5-7 Clinicilor St., 400006 Cluj-Napoca, Romania; 3 Integrated Electron Microscopy Laboratory, National Institute for Research and Development of Isotopic and Molecular Technologies, 67-103 Donat St., 400293 Cluj-Napoca, Romania; 4 RDI Laboratory of Applied Raman Spectroscopy, RDI Institute of Applied Natural Sciences (IRDI-ANS), Babeş-Bolyai University, Fântânele 42, 400293 Cluj-Napoca, Romania; 5 Department of Pharmaceutical Physics-Biophysics, Faculty of Pharmacy, Iuliu Hatieganu University of Medicine and Pharmacy, 6 Pasteur St., 400349 Cluj-Napoca, Romania

**Keywords:** zinc–cobalt ferrite nanoparticles, Raman measurements, magnetic properties

## Abstract

Co_1−*x*_Zn*_x_*Fe_2_O_4_ nanoparticles (0 ≤ *x* ≤ 1) have been synthesized via a green sol–gel combustion method. The prepared samples were studied using X-ray diffraction measurements (XRD), transmission electron microscopy (TEM), Raman, and magnetic measurements. All samples were found to be single phases and have a cubic *Fd-3m* structure. EDS analysis confirmed the presence of cobalt, zinc, iron, and oxygen in all studied samples. Raman spectra clearly show that Zn ions are preferentially located in T sites for low Zn concentrations. Due to their high crystallinity, the nanoparticles show high values of the magnetization, which increases with the Zn content for *x* < 0.5. The magnetic properties are discussed based on Raman results. Co ferrite doped with 30% of Zn produced the largest SAR values, which increase linearly from 148 to 840 W/g_MNPs_ as the H is increased from 20 to 60 kA/m.

## 1. Introduction

Magnetic materials with nanometer crystalline sizes are the focus of a large number of researchers due to their interesting changes on physical properties compared to that of their bulk correspondent. The physical properties of these materials can be easily tuned and depend strongly on the chemical composition, crystal structure, shape, and size of the nanoparticle, as well as the cation distribution [1,2]. Among these, cobalt ferrite with or without substitution on cobalt site is technologically important for applications in different fields: biomedicine, anti-cancer drugs, cellular therapy, active components in ferrofluids, magnetic refrigeration, information and energy storage media, sensors, switching devices, high frequency electric devices, recording heads, catalysis, magnetic cell separation, hyperthermia, antibacterial agents, permanent magnets, medical imaging, etc. [3,4,5,6,7,8,9,10,11,12,13,14,15].

CoFe_2_O_4_ presents high coercivity, high cubic magneto crystalline anisotropy, moderate saturation magnetization, large magnetostrictive coefficient, and low toxicity [16,17,18,19,20,21]. On the other hand, cobalt ferrite exhibit high mechanical stiffness and chemical stability.

The CoFe_2_O_4_, or (Co_1−*x*_Fe_*x*_)_T_(Co_*x*_Fe_2−*x*_)_O_O_4_ structure, where *x* is the inversion parameter, presents a variable occupancy of tetrahedral (T) and octahedral sites (O). The structure changes from a normal spinel (*x* = 0) to an inverse spinel type (*x* = 1). In bulk state, the CoFe_2_O_4_ ferrite has a predominantly inverse-type spinel structure and crystallizes in a fcc-type lattice, space group $Fm\bar{3}m$. CoFe_2_O_4_ is ferrimagnetically ordered, with the magnetic moments of the atoms situated in T and O sublattices being antiparallelly aligned. Depending on the preparation method and the annealing temperature, Co^2+^ ions could resize in octahedral sites and will strongly influence the structural, magnetic, and electric properties [22,23,24].

ZnFe_2_O_4_ crystallize in a normal spinel structure with the Zn^2+^ ions located mainly in the T sites and Fe^3+^ ions located in O sites [16,25]. It was reported that the cobalt ferrite physical properties can be adjusted by the substitution with magnetic or non-magnetic ions. Li et al. have reported that the saturation magnetization of Co_1−*x*_Zn_*x*_Fe_2_O_4_ (prepared using the sol–gel method) increase when Zn ions concentration increases up to *x* = 0.4, followed by a large decrease for *x* > 0.5 [26]. The initial increase in the magnetization was explained by the preferential occupation of T sites by Zn^2+^. When the zinc concentration increases more, the exchange interactions between Fe^3+^ ions decrease due to the lack of magnetic neighbors and the saturation magnetization decreases. Later, Slatineanu et al. reported that on the same system, prepared by chemical co-precipitation method, the compound with *x* = 0.2 is optimum considering magnetic and dielectric properties [27]. Atif et al. has shown that the maximum value of the saturation magnetization on Co_1−*x*_Zn_*x*_Fe_2_O_4_ prepared by sol–gel method was obtained for *x* = 0.4 and was explained by the preferential occupation of T sites by Zn ions [28]. Tanaka et al. have prepared zinc ferrites by precipitation of the rapidly quenched oxides [29]. Small amounts of Zn and magnetite was found in the prepared samples. From Zn K-edge EXAFS studies, it was shown that some of the Zn^2+^ ions occupy octahedral sites together with the occupancy of tetrahedral sites by the Fe^3+^ ions.

Previously, we have studied the structural and magnetic properties of CoFe_2_O_4_ nanoparticles and CoFe_2_O_4_ @SiO_2_ @Au nanocomposites designed for magnetoplasmonic applications [30,31]. To check the Zn ion occupancy in the cobalt ferrite structure and to improve the values of saturation magnetization, we have prepared cobalt ferrites with Zn substitution at Co sites in very large concentration ranges. In this paper, we present our results on the influence of Zn on the structural and physical properties of cobalt ferrite. The Co_1−*x*_Zn_*x*_Fe_2_O_4_ nanoparticles have been synthesized via a green sol–gel combustion method. Chemical reduction, often used to produce nanoferrites, utilizes organic solvents. Risk-free chemicals, environmentally mild solvents and feasible materials are key factors to the application of green strategies [32,33]. The green synthesis of nanoparticles denotes a development above other methods, since it is simpler, easy to use, and the obtained nanoferrites are often more stable. The prepared samples were studied using X-ray diffraction measurements (XRD), transmission electron microscopy (TEM), scanning electron microscopy (SEM), Raman, and magnetic measurements. Raman spectra clearly show that Zn ions are preferentially located in T sites for low Zn concentrations. The magnetic properties are discussed based on Raman results.

## 2. Materials and Methods

### 2.1. Samples Preparation

Co_1−*x*_Zn_*x*_Fe_2_O_4_ (*x* = 0, 0.05, 0.1, 0.2, 0.3, 0.4, 0.5, 0.6, 0.7, 0.8, 0.9, 1) nanoparticles were synthesized via a green, sucrose, and pectin-based, sol–gel combustion method. The synthesis can be considered green as both the poly-condensation and chelating agents were molecules of vegetal origin. The sucrose used was extracted from sugar beets and the pectin used for the synthesis was extracted from citrus fruits. The reagents, Fe(NO_3_)_3_⸱9H_2_O (Iron(III) nitrate nonahydrate), Co(NO_3_)_2_⸱6H_2_O (Cobalt nitrate hexahydrate 97.7%), Zn(NO_3_)_2_ (Zinc nitrate hexahydrate 98%), were purchased from Alfa Aesar. All calculations were performed for 2 mmols of nanoparticles. Stoichiometric quantities of each precursor were weighed and dissolved in Milli-Q water under vigorous magnetic stirring at 60 °C. After 1 h, 14.9 mmols sucrose (≈5 g) was added to each solution. After the full homogenization, the pH of the solutions was lowered to approximately 2 using a 65% HNO_3_ solution. For each solution, one gram of pectin was weighed and slowly added to the mixture under magnetic stirring. This was performed in order to stop the pectin from agglomerating. After 20 more minutes of further stirring, the solutions were poured in ceramic capsules and placed on a sand bath at 240 °C for 24 h in order to evaporate the water and obtain the gel. The spongy, dried gel was then annealed in air at 700 °C for 2 h in order to decompose the organic part of the gel. A fine nanopowder was obtained.

### 2.2. Characterization

The crystal structure and crystallite sizes of Co_1−*x*_Zn_*x*_Fe_2_O_4_ nanoparticles were determined by XRD measurements, performed at ambient temperature, with a Bruker D8 Advance diffractometer. The intensities were measured from 20° to 80° in continuous mode with a step size of 0.03° and a counting rate of 5 s per scanning step. The lattice parameters were obtained by Rietveld refinement of XRD patterns using FullProf Suite Software [34]. The instrumental resolution function (IRF) was evaluated by fitting the diffraction pattern of an LaB6 NIST standard and recorded under the same experimental conditions as those used for measuring the ferrite nanoparticles, and the IRF data file was provided to the program in order to allow subsequent refinement of the diffraction pattern of the samples. The refinement was performed based on the Thompson–Cox–Hastings pseudo-Voigt functions for peaks profile and the refined parameters were the lattice parameter, oxygen position, zero-shift correction, background parameters, isotropic temperature factor, and peak shape parameters. The crystallite sizes were estimated using the Debye–Scherrer equation:

(1) $$D=\frac{k\lambda }{\beta \cos\theta }$$

where *β* is the peak full width at half maximum (in radians) at the observed peak angle *θ*, k is the crystallite shape factor (was considered 0.9), and λ is the X-ray wavelength.

The morphology of the synthesized nanoparticles was investigated by transmission electron microscopy (TEM) and scanning electron microscopy (SEM) using a Hitachi HD2700 CFEG STEM at 200 kV with secondary electron imaging capability. The energy dispersive X-ray spectroscopy (EDS) measurements were performed in order to analyze the composition of the prepared nanocomposites.

Dry nanoparticles were resuspended in 0.5 mL of distilled water and sonicated for 10 s in an ultrasonic bath to obtain a suspension. From here, 10 μL were pipetted onto a quartz plate enabling firm and localized adhesion of the nanoparticles. Micro-Raman analysis was done after complete water evaporation. A Renishaw InVia Reflex confocal Raman microscope was used for structural analysis, using 100× objective (NA 0.9) and He-Ne laser emitting at 632.8 nm (full power 17 mW). The spectra were acquired from the nanoparticle aggregates about 5 μm large, with 60 s integration, 5 additive scans per spectrum, and 1.7 mW laser power. These conditions allowed the acquisition of Raman signal of sufficient intensity without causing phase transitions or fluorescence from the sample due to laser heat input [35]. At least 3 spectra were acquired from different points of each sample. Raman spectra were processed in Origin 8.5 software.

Magnetic measurements were performed in the 4.2–300 K temperature range and external magnetic fields up to 10 T by using a vibrating sample magnetometer from Cryogenic Limited London. The heating efficiency was evaluated using a commercially available magnetic hyperthermia system, the Easy Heat 0224 from Ambrell (Scottsville, NY, USA), equipped with an optical fiber temperature sensor (0.1 °C accuracy), providing alternating magnetic field (AMF) of fixed frequency (355 kHz) and variable amplitude (5–65 kA/m). Details about specific absorption rate (SAR) calculations are provided in the Supplementary Materials (Section S1).

## 3. Results

### 3.1. Morphology and Crystal Structure

**Figure 1:**
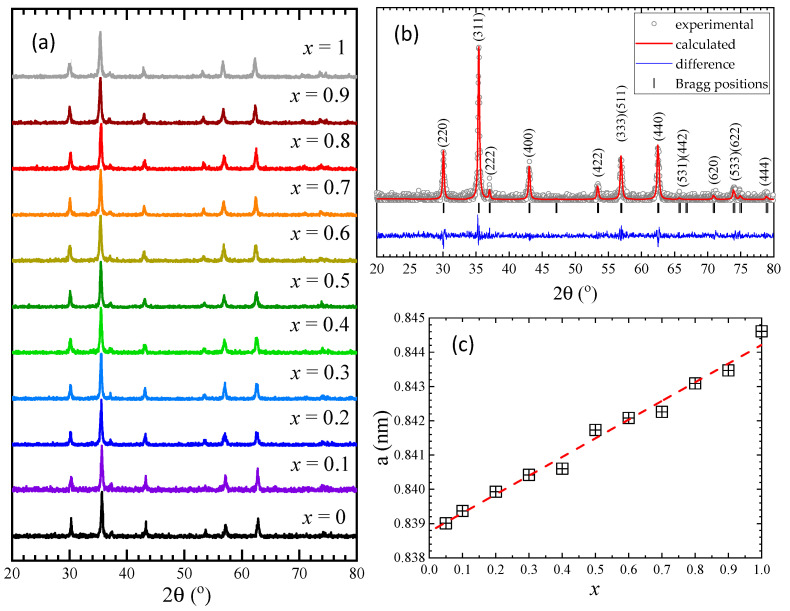
(**a**) XRD patterns at room temperature of Co_1−*x*_Zn_*x*_Fe_2_O_4_ nanoparticles, (**b**) Rietveld refinement results for Co_0.7_Zn_0.3_Fe_2_O_4_, (**c**) The lattice parameter/unit cell volume dependence with Zn concentration for Co_1−*x*_Zn_*x*_Fe_2_O_4_.

The X-ray diffraction (XRD) measurements (Figure 1a) indicate that all samples are single phases and have a cubic *Fd-3m* structure. In order to determine the lattice parameters, Rietveld analysis was performed for all samples using FullProf software [34], as shown for Co_0.7_Zn_0.3_Fe_2_O_4_ in Figure 1b. The Rietveld analysis shows an almost linear increase in the lattice parameter/unit cell volume with the Zn content (Table 1), as expected considering the larger ionic radius of Zn, as compared with that of Co ions. The lattice parameter values and their Zn content dependence is close to that previously reported by other groups in a narrower concentration range [27,28,29] and opposite to ref. [26], where a decrease was shown with a minimum at *x* = 0.3 followed by a nonlinear increase with Zn content. The crystallinity of our samples is probably higher than in that case.

The crystallite sizes, calculated using the Debye–Scherrer formula after subtracting the instrumental peak broadening, were found to be between 30 nm and 40 nm for all investigated Co_1−*x*_Zn_*x*_Fe_2_O_4_ samples.

**Figure 2:**
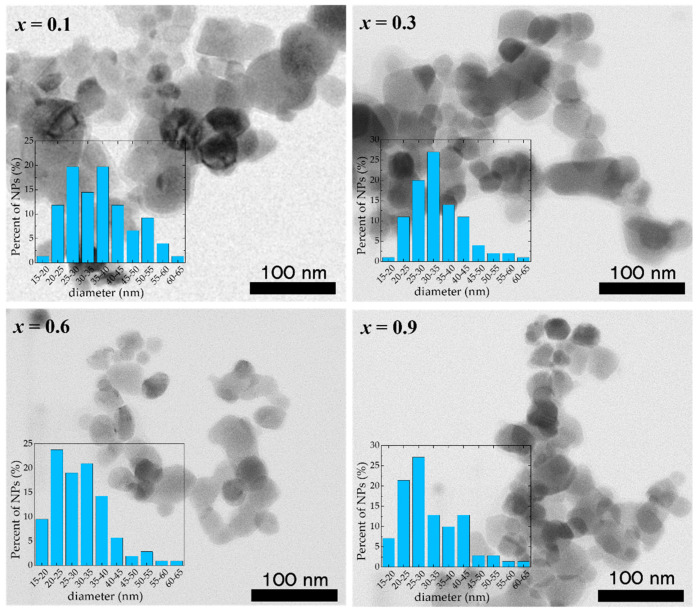
TEM images of Co_1−*x*_Zn_*x*_Fe_2_O_4_ nanoparticles.

The TEM investigations of the Co_1−*x*_Zn_*x*_Fe_2_O_4_ samples reveal that well-defined nanoparticles tend to agglomerate due to their significant magnetic interaction that competes with the much weaker electrostatic repulsion (Figure 2). The analysis of the TEM images shows the presence of nanoparticles of sizes between 15 nm and 70 nm, with a mean diameter close to the values obtained from XRD (Table 2), indicating that most of the Co_1−*x*_Zn_*x*_Fe_2_O_4_ nanoparticles are single crystals and have high crystallinity. One can see that the nanoparticles are highly polydisperse and the error bars of the dimensions obtained from TEM measurements indicate that the nanoparticles’ average diameters could be higher compared with the mean diameter obtained from XRD data.

**Figure 3:**
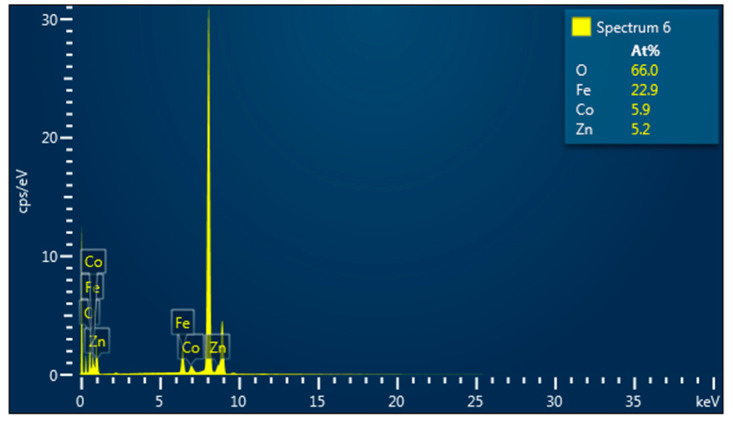
EDS spectra for Co_0.4_Zn_0.6_Fe_2_O_4_ nanoparticles.

**Figure 4:**
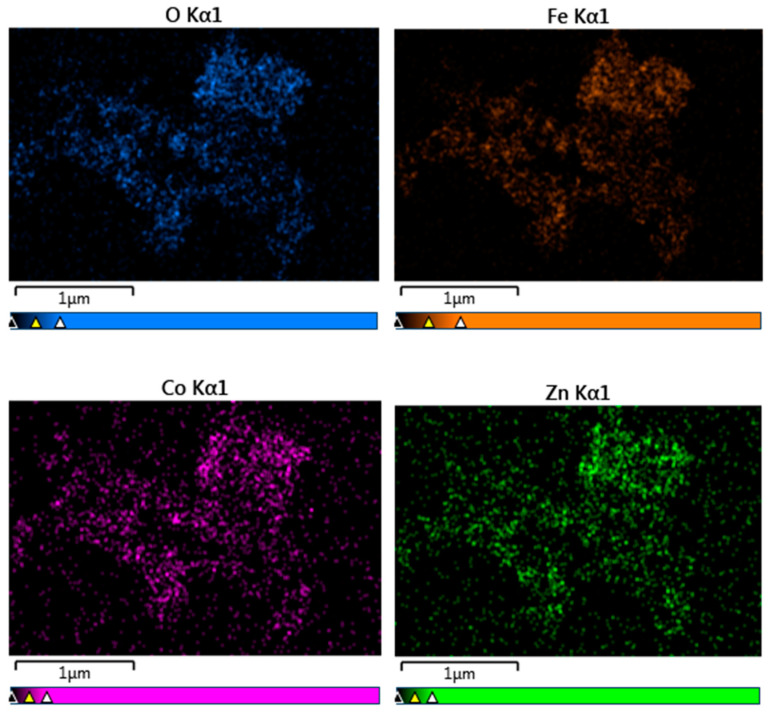
Elemental mapping of Co_0.4_Zn_0.6_Fe_2_O_4_ nanoparticles.

Elemental analysis by EDS confirmed the presence of Co, Fe, Zn, and O elements in all studied samples. An example of the EDS spectra for Co_0.4_Zn_0.6_Fe_2_O_4_ nanoparticles is given in Figure 3. The EDS spectra indicate that there are no other impurities present in the nanoparticles. The elemental mapping for the sample with *x* = 0.6 is shown in Figure 4. One can see that the Co, Zn, Fe, and O elements are almost homogeneously distributed across the selected zone of the samples investigated. Similar results were obtained for all studied samples. The elemental abundances for the studied samples are listed in Table 2. These data show that the concentrations of Zn, Co, and Fe elements in the resulting product are in a close agreement with the expected stoichiometric ratio.

### 3.2. Raman Spectra

Raman spectroscopy is a useful tool in analyzing the cation distribution in spinels. The cubic inverse/mixed ferrite structure gives rise to 39 normal vibrational modes, out of which 5 are Raman active: A_1g_, E_g_, and 3T_2g_ [36,37,38,39]. The Raman band configuration of iron oxides [35], face-centered cubic structure ferrites with *Fd-3m* symmetry, such as magnetite and maghemite [40] and doped ferrites [41,42,43,44,45], has been explored by several previous studies. Above 600 cm^−^^1^, the observed A_1g_ Raman modes are due to the symmetric metal-O stretching vibrations at tetrahedral sites, while the lower T_2g_ and E_g_ frequency modes are associated with metal-O vibrations at octahedral sites [46].

**Figure 5:**
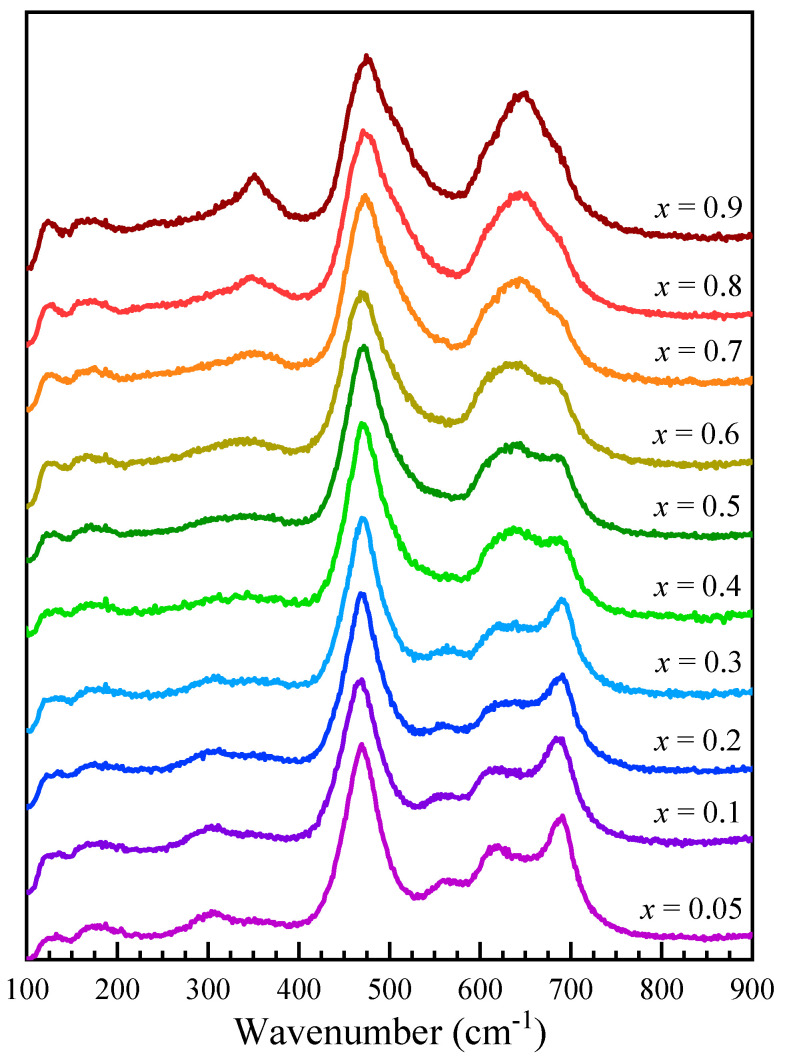
Raman spectra of Co_1−*x*_Zn_*x*_Fe_2_O_4_ nanoparticles.

Figure 5 shows the Raman spectra recorded for Co_1−*x*_Zn*_x_*Fe_2_O_4_ nanoparticles as 0.05 ≤ *x* ≤ 0.9. The T_2g_(1) band is centered at about 180 cm^−^^1^, slightly lower than observed in magnetite (196 cm^−^^1^) and higher than in Mn-Zn-doped ferrites (170 cm^−^^1^) [44]. Above 200 cm^−^^1^, the Raman spectra of Co_1−*x*_Zn*_x_*Fe_2_O_4_ nanoparticles show several modes, located at about 310 cm^−^^1^, 350 cm^−^^1^, 470 cm^−^^1^, 560 cm^−^^1^, 610 cm^−^^1^, 650 cm^−^^1^, and 690 cm^−^^1^, that change in intensity with the Zn content. In order to assign the different contributions to the recorded spectra, the modes associated with Co, Zn, and Fe ions located in the tetrahedral and/or octahedral sites have been considered. In cobalt ferrite, the A_1g_ modes located at about 620 cm^−^^1^ and 695 cm^−^^1^ were found to correspond to Co and Fe ions located at the tetrahedral site, while the T_2g_(3) modes, corresponding to the Fe and Co ions at the octahedral sites, are located at about 470 cm^−1^ and 575 cm^−^^1^ [41]. A detailed analysis of the Raman spectra in ZnFe_2_O_4_ revealed that the A_1g_ broad peak around 650 cm^−^^1^ is the result of the overlapping of two signals centered around 641 cm^−1^ and 685 cm^−1^, which correspond to the modes inside ZnO_4_ and FeO_4_ units, while the T_2g_(3) modes due to the Fe and Zn ions are located at about 470 cm^−1^ and 510 cm^−1^ [46].

Raman spectroscopy analysis and spectra deconvolution was conducted primarily to explore if cation distribution between the tetrahedral and octahedral sites can be quantified. The movement of additional cation types to tetrahedral sites adds new components to the A_1g_ band, the position of which is loosely correlated to respective ionic radius of the dopant [44]. Tetrahedrally coordinated zinc within ferrites exhibits a band in the 630–650 cm^−^^1^ range [44,45,46], well separated from the Co–O and Fe–O bands. Indeed, the fits show the three A_1g_ components at 605–610 cm^−^^1^ representing Co–O, at 635–650 cm^−^^1^ representing Zn–O, and at 680–690 cm^−^^1^ representing Fe–O vibrations.

In order to analyze the cation distribution, the Raman spectra of Co_1−*x*_Zn_*x*_Fe_2_O_4_ nanoparticles were fitted with six components in the region between 380 cm^−^^1^ and 800 cm^−^^1^, which correspond to the Fe, Zn, and Co ions located in the tetrahedral and octahedral sites, with widths between 40 cm^−^^1^ and 70 cm^−^^1^. The fitting results indicate position shifts with only up to ± 10 cm^−^^1^, indicating the preservation of the general Co ferrite phase (Table 3).

**Figure 6:**
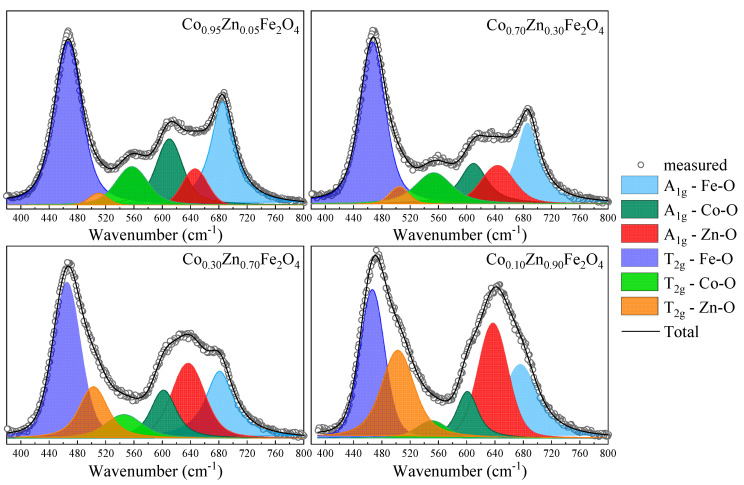
Fitting results for Raman spectra of Co_1−*x*_Zn*_x_*Fe_2_O_4_ nanoparticles.

The deconvolution of the Raman spectra, as shown for selected spectra in Figure 6, reveal that for low Zn content, the Zn ions substitute the Co^2+^ ions in the tetrahedral sites, as indicated by the decrease in the intensity of the peak located at about 610 cm^−^^1^, associated with the A_1g_ mode of the CoO_4_ units. As the Zn content increases for *x* > 0.4, the contribution from the Zn ions in the octahedral sites at about 510 cm^−^^1^ increases. The changes in the intensities of the Raman modes attributed to the Fe ions in the tetrahedral sites (~680 cm^−^^1^) and octahedral sites (~470 cm^−^^1^) indicate changes in the inversion degree with the Co substitution by Zn.

### 3.3. Magnetic Measurements

**Figure 7:**
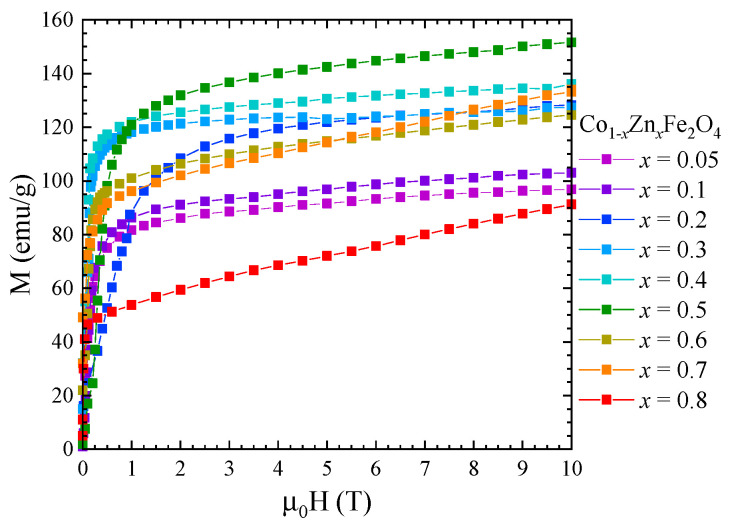
Magnetization isotherms of Co_1−*x*_Zn*_x_*Fe_2_O_4_ nanoparticles recorded at 4 K.

The saturation magnetization, $M_{s}$, at 4 K of Co_1−*x*_Zn*_x_*Fe_2_O_4_ nanoparticles was determined from magnetic measurements in fields up to 10 T (Figure 7) using the approach to saturation law:

$$M=M_{s}\left(1-\frac{a}{H}\right)+\chi_{0}H$$

where *a* is the coefficient of magnetic hardness and $\chi_{0}$ is the Pauli type contribution.

**Figure 8:**
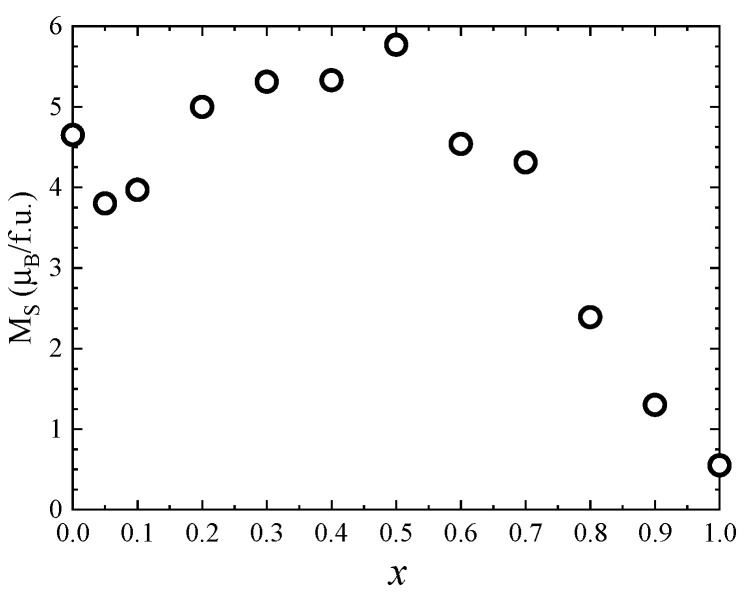
Saturation magnetization of Co_1−*x*_Zn_*x*_Fe_2_O_4_ nanoparticles.

One can see that the saturation magnetization increases at the beginning when Zn concentration increases with a maximum at *x* = 0.5, followed by a strong decrease in the higher Zn content—Figure 8. Previous studies reported a maximum value of the saturation magnetization for *x* = 0.2 [27] and *x* = 0.4 [26,28]. The fact that in our case the maximum saturation magnetization of about 5.8 µ_B_ was obtained at higher Zn content can be explained by the high quality and crystallinity of our samples. The initial increase in saturation magnetization with Zn doping level is attributed to the preferential substitution of Co^2+^ by nonmagnetic Zn^2+^ ions in the tetrahedral sites, in agreement with the Raman data. For *x* ≥ 0.5, the saturation magnetization decreases, which can be explained by the substitution of Co ions in the octahedral coordination, as indicated by the Raman spectra. The fact that there is no linear dependence of the saturation magnetization with the Zn content for either *x* < 0.5 or *x* > 0.5 is consequence of the changes in inversion degree with the concentration.

**Figure 9:**
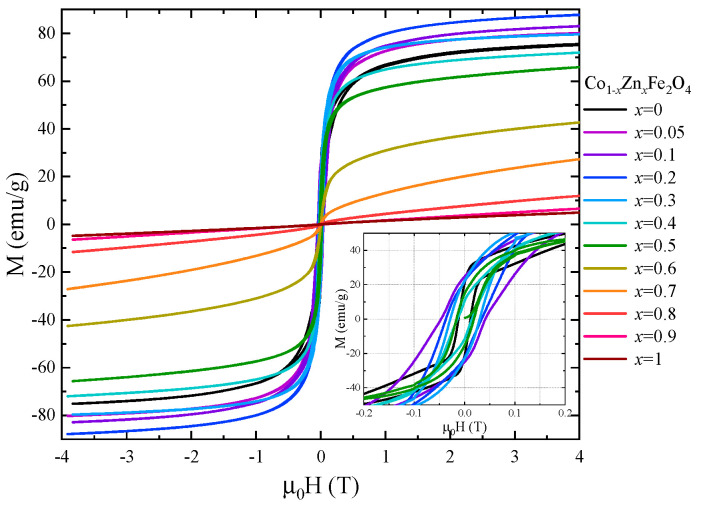
Hysteresis loops recorded at room temperature for Co_1−*x*_Zn*_x_*Fe_2_O_4_ nanoparticles.

**Figure 10:**
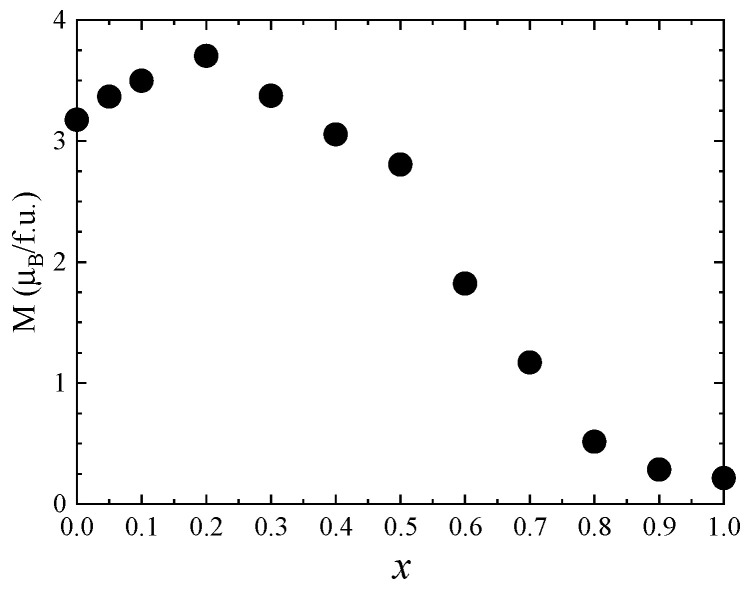
The magnetization values in 4 T of Co_1−*x*_Zn*_x_*Fe_2_O_4_ nanoparticles at 300 K.

The magnetic properties of Co_1−*x*_Zn_*x*_Fe_2_O_4_ nanoparticles at room temperature were also investigated by recording the hysteresis loops between −4 T and 4 T (Figure 9). The coercive field has a maximum value of 0.05 T for *x* = 0.1 and decreases with the Zn content for *x* > 0.1. The magnetization values at 300 K in 4 T (Figure 10) show a similar behavior to that of the spontaneous magnetization at 4 K.

**Figure 11:**
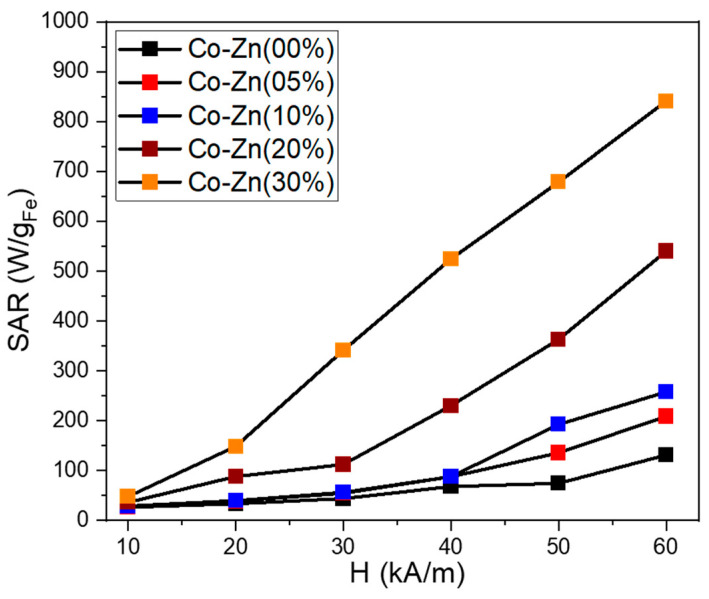
Specific absorption rate (SAR) dependence on the magnetic field amplitude (H) for the five ferrite samples dispersed in water at a concentration of 1 mg_MNPs_/mL.

The magnetically induced heating capabilities of five samples were investigated in water at one certain concentration of 1 mg_MNPs_/mL. The Box–Lucas function was employed to fit the heating curves and the resulting parameters were used to evaluate the specific absorption rate (SAR) in watts per unit mass of MNPs (W/g_MNPs_). The SAR values were plotted as a function of the amplitude (H) of the applied AMF, ranging from 10 kA/m to 60 kA/m (step of 10 kA/m) at a fixed frequency of 355 kHz. The contribution from pure water at each H was measured and subtracted as the background. Figure 11 summarizes the obtained mean SAR values for the analyzed ferrite particles. The Co ferrites particles without Zn content present low SAR values compared with other studies [47,48,49]. At the H of 10 kA/m the SAR is 26 W/g_MNPs_, which increases with the increasing H, reaching a value of 113 W/g_MNPs_ for the highest H of 60 kA/m used in the study. A Zn doping of 5% and 10% do not significantly influence the heating performances of Co ferrite particles, specifically for H between 10 and 40 kA/m. A clear increase in SAR values with Zn doping is observed only for the H of 50 and 60 kA/m. Excepting the field H = 10 kA/m, for the rest values of H (20–60 kA/m), the SAR values increase considerably for Co ferrite bearing a Zn doping of 20%. A SAR of 88 and 112 W/g_MNPs_ is recorded at 20 and 30 kA/m, respectively, which is double that of the previous three samples. Starting with 40 kA/m, the SAR increment is faster, going up to 540 W/g_MNPs_ for the highest H of 60 kA/m. The SAR evolution with H is typical of a hard sample [47]. It is worth mentioning that, for each H value, the SAR increased as the Zn doping level was increased. This behavior can be explained by considering the similar dependence of saturation magnetization on the Zn content [50].

The magnetic characterization revealed that by further increasing the Zn content to 30%, both the M_s_ and H_c_ decreased. However, these particles exhibit the largest SAR values among all five samples. With respect to the previous sample (20% Zn content), the SAR values are enhanced by a factor that increase from 1.3 to 3 as the H is swept from 10 to 60 kA/m. It can be observed that the SAR values increase linearly from 148 to 840 W/g_MNPs_ as the H is increased from 20 to 60 kA/m. This peculiar dependence of SAR with the applied H is in accordance with the evolution of magnetization that does not saturate and exhibits a slow positive slope as the external static magnetic field is increased. Due to its very good heating capabilities, this sample represents a good candidate for future in vitro magnetic hyperthermia experiments.

## 4. Conclusions

Highly crystalline Co_1−*x*_Zn_*x*_Fe_2_O_4_ nanoparticles (0 ≤ *x* ≤ 1), with average sizes between 30 nm and 40 nm, have been synthesized via a green sol–gel combustion method, as shown by XRD and TEM structural investigations. Elemental analysis by EDS confirmed the presence of Co, Fe, Zn, and O elements in all studied samples. Raman spectroscopy was employed in analyzing the cation distribution, since the Raman modes corresponding to the Fe, Zn, and Co ions located in the tetrahedral and octahedral sites are well separated in the region between 380 cm^−1^ and 800 cm^−1^. The deconvolution of the spectra indicates that for low Zn content (*x* < 0.4), the Zn ions preferentially substitute the Co^2+^ ions in the tetrahedral sites. Additionally, the changes in the intensities of the Raman modes attributed to the Fe ions in the tetrahedral sites and octahedral sites suggest changes in the inversion degree with the Co substitution by Zn. The magnetic properties of the investigated nanoparticles reflect the structural changes. Due to their high crystallinity, the nanoparticles show high values of the magnetization, which increases with the Zn content for *x* < 0.5, which can be explained by the substitution of Co ions in the tetrahedral sites. For higher Zn concentration, as the Zn ions replace Co in the octahedral sites, the total magnetization of the Co_1−*x*_Zn_*x*_Fe_2_O_4_ nanoparticles decreases.

The increase in the Zn doping level led to an increase in the heating performances of Co ferrite. With respect to the undoped sample, an SAR enhancement by a factor between 4.5 and 9 over the H range between 20 and 60 kA/m was detected for 30% Zn content.

In the future, we propose to do in vitro hyperthermia experiments and to cover these nanoparticles with a BaTiO_3_ shell in order to test the possibility of controlling the shell electric polarization with the magnetic field while keeping in mind possible applications in cancer treatment.

## Figures and Tables

**Table 1:** Structural parameters and Rietveld agreement factors, such as weighted profile factor (R_wp_), expected factor (R_exp_), and goodness-of-fit (χ^2^), of Co_1−*x*_Zn_*x*_Fe_2_O_4_ nanoparticles.

*x*	Lattice Parameter a (nm)	χ^2^	R_wp_	R_exp_
0.05	0.8390 (1)	1.04	7.08	6.95
0.1	0.8393 (7)	1.03	7.27	7.15
0.2	0.8399 (2)	0.934	5.90	6.11
0.3	0.8404 (1)	0.937	5.71	5.91
0.4	0.8405 (5)	0.994	5.95	5.97
0.5	0.8418 (0)	0.948	5.56	5.72
0.6	0.8421 (0)	1.00	5.65	5.64
0.7	0.8422 (4)	1.03	5.47	5.39
0.8	0.8430 (9)	0.988	5.75	5.79
0.9	0.8434 (7)	0.970	5.34	5.42
1	0.8446 (1)	0.975	5.53	5.60

**Table 2:** Average Co_1−*x*_Zn_*x*_Fe_2_O_4_ nanoparticles’ size determined from XRD and TEM investigations, and atomic elemental composition theoretically calculated and determined from EDS measurements.

*x*	Mean Diameter (nm)		Atomic Abundance (Atoms %)							
	XRD	TEM	Co th.	Co exp.	Zn th.	Zn e.	Fe th.	Fe exp.	O th.	O exp.
0.1	35 ± 2	38 ± 11	12.8	11.5	1.4	1.1	28.5	21.3	57.3	66.1
0.3	36 ± 2	35 ± 9	10.0	9.0	4.2	1.6	28.5	21.4	57.3	68.0
0.6	31 ± 1	31 ± 10	5.7	5.9	8.5	5.2	28.5	22.9	57.3	66.0
0.9	32 ± 1	31 ± 10	1.4	1.9	12.8	9.0	28.5	23.6	57.3	65.5

**Table 3:** Fitting results of the main Raman modes for Co_1−*x*_Zn_*x*_Fe_2_O_4_ nanoparticles.

*x*	T_2g_		A_1g_	
	Energy (cm^−1^)	FWHM (cm^−1^)	Energy (cm^−1^)	FWHM (cm^−1^)
0.05	467 ± 1 (Fe–O)	57 ± 5	610 ± 1 (Co–O)	54 ± 5
	510 ± 1 (Zn–O)	43 ± 5	647 ± 1 (Zn–O)	46 ± 5
	557 ± 1 (Co–O)	62 ± 5	686 ± 1 (Fe–O)	47 ± 5
0.1	465 ± 1 (Fe–O)	56 ± 5	604 ± 1 (Co–O)	54 ± 5
	506 ± 1 (Zn–O)	42 ± 5	641 ± 1 (Zn–O)	69 ± 5
	551 ± 1 (Co–O)	51 ± 5	683 ± 1 (Fe–O)	51 ± 5
0.2	466 ± 1 (Fe–O)	53 ± 5	607 ± 1 (Co–O)	52 ± 5
	503 ± 1 (Zn–O)	44 ± 5	642 ± 1 (Zn–O)	68 ± 5
	554 ± 1 (Co–O)	69 ± 5	686 ± 1 (Fe–O)	44 ± 5
0.3	467 ± 1 (Fe–O)	52 ± 5	610 ± 1 (Co–O)	56 ± 5
	505 ± 1 (Zn–O)	37 ± 5	644 ± 1 (Zn–O)	62 ± 5
	555 ± 1 (Co–O)	68 ± 5	687 ± 1 (Fe–O)	41 ± 5
0.4	468 ± 1 (Fe–O)	51 ± 5	608 ± 1 (Co–O)	45 ± 5
	503 ± 1 (Zn–O)	61 ± 5	643 ± 1 (Zn–O)	55 ± 5
	558 ± 1 (Co–O)	69 ± 5	686 ± 1 (Fe–O)	48 ± 5
0.5	465 ± 1 (Fe–O)	53 ± 5	604 ± 1 (Co–O)	45 ± 5
	504 ± 1 (Zn–O)	64 ± 5	639 ± 1 (Zn–O)	64 ± 5
	554 ± 1 (Co–O)	66 ± 5	683 ± 1 (Fe–O)	46 ± 5
0.6	465 ± 1 (Fe–O)	52 ± 5	602 ± 1 (Co–O)	53 ± 5
	503 ± 1 (Zn–O)	59 ± 5	638 ± 1 (Zn–O)	64 ± 5
	547 ± 1 (Co–O)	67 ± 5	682 ± 1 (Fe–O)	49 ± 5
0.7	468 ± 1 (Fe–O)	45 ± 5	605 ± 1 (Co–O)	47 ± 5
	505 ± 1 (Zn–O)	68 ± 5	642 ± 1 (Zn–O)	56 ± 5
	552 ± 1 (Co–O)	45 ± 5	684 ± 1 (Fe–O)	48 ± 5
0.8	468 ± 1 (Fe–O)	47 ± 5	603 ± 1 (Co–O)	42 ± 5
	505 ± 1 (Zn–O)	68 ± 5	640 ± 1 (Zn–O)	67 ± 5
	550 ± 1 (Co–O)	63 ± 5	682 ± 1 (Fe–O)	48 ± 5
0.9	467 ± 1 (Fe–O)	46 ± 5	602 ± 1 (Co–O)	44 ± 5
	504 ± 1 (Zn–O)	65 ± 5	639 ± 1 (Zn–O)	57 ± 5
	552 ± 1 (Co–O)	62 ± 5	682 ± 1 (Fe–O)	61 ± 5

## Data Availability

Not applicable.

## Supplementary Materials

The following are available online at https://www.mdpi.com/article/10.3390/nano13010189/s1, the details of magnetic measurements and specific absorption rate (SAR) calculations are provided in the supplementary materials. Reference [51] are cited in the supplementary materials.
