# Supplementary material for: The Influence of Zn Substitution on Physical Properties of CoFe2O4 Nanoparticles
Source: Nanomaterials (Basel). 2022 Dec 31;13(1):189. doi: 10.3390/nano13010189 (PMC9823853; doi:10.3390/nano13010189)
Supplement: Supplementary file 1 [file nanomaterials-13-00189-s001.zip › nanomaterials-2095334-supplementary.pdf]

Supplementary Material

The Influence of Zn Substitution on Physical Properties of $CoFe_2O_4$ Nanoparticles

Adam Szatmari [1], Rareș Bortnic [1], Gabriela Souca [1], Răzvan Hirian [1], Lucian Barbu-Tudoran [2,3], Fran Nekvapil [1,4], Cristian Iacovita [5], Emil Burzo [1], Roxana Dudric [1,*] and Romulus Tetean [1,*]

1 Faculty of Physics, "Babes Bolyai" University, Kogalniceanu 1, 400084 Cluj-Napoca, Romania
2 Electron Microscopy Center "Prof. C. Craciun", Faculty of Biology & Geology, "Babes-Bolyai" University, 5-7 Clinicilor St., 400006 Cluj-Napoca, Romania
3 Integrated Electron Microscopy Laboratory, National Institute for Research and Development of Isotopic and Molecular Technologies, 67-103 Donat St., 400293 Cluj-Napoca, Romania
4 RDI Laboratory of Applied Raman Spectroscopy, RDI Institute of Applied Natural Sciences (IRDI-ANS), Babeş-Bolyai University, Fântânele 42, 400293 Cluj-Napoca, Romania
5 Department of Pharmaceutical Physics-Biophysics, Faculty of Pharmacy, Iuliu Hatieganu University of Medicine and Pharmacy, 6 Pasteur St., 400349 Cluj-Napoca, Romania
* Correspondence: roxana.dudric@ubbcluj.ro (R.D.); romulus.tetean@ubbcluj.ro (R.T.); Tel.: +40-741033325 (R.T.)

S1. Magnetic hyperthermia:

The EasyHeat 0224 power supply station (Ambrell, Scottsville, NY, USA) is equipped with an eight-turn coil with an internal diameter of 2.5 mm and a total length of 40 mm. The inductance of the coil was calculated from its geometry and was determined as 10^{-6} H. The effective frequency and voltage values on the coil were monitored with a digital oscilloscope PeakTech 1170 (PeakTech Prüf-und Messtechnik GmbH, Ahrensburg, Germany) operating up to 250 MHz, and for the specified coil was 355 kHz. The H calibration was performed by using a copper wire with a 10 mm diameter, surrounding the vial in which the samples were introduced as a magnetic probe, and measuring the induced electromotive force by using the oscilloscope, as described in detail in our previous work [1]. The samples consisted of a 0.5 mL volume of Co ferrite suspended in water. The temperature was assessed using a optic fiber, placed in the middle of the sample, to provide the temperature values at one second intervals.

The specific absorption rate (SAR) is defined as the heat released from a suspension of MNPs in unit time reported to the mass of iron content. It was used to quantify the heat performance of MNPs. For reliable determination of SAR, the temperature change ΔT versus time curves - where $\Delta T = T(t) - T_0$; T(t) is the temperature at time t and T_0 = 37 °C - have been fitted with the Box-Lucas equation (Figure S1):

$$\Delta T = \frac{S_m}{k}\left(1 - e^{-k(t-t_0)}\right) \quad \text{(S1)}$$

where the fitting parameters S_m and k are the initial slope of the heating curve and the constant describing the cooling rate, respectively. Thus, SAR can be calculated as:

$$\text{SAR} = \frac{c\, m\, S_m}{m_{MNPs}} \quad \text{(S2)}$$

where c is the specific heat of the colloid (in our case was approximated with the specific heat of water: $c = 4186\, J/kgK$ the MNPs contribution to the specific heat being negligible), $m = \rho V$ is the mass of colloid, taken as the product between the density ($\rho_{water} = 0,997\, g/cm^3$ at 298K) and the volume. Prior to each measurements the sample has been sonicated for 30 seconds to assure a good colloidal dispersion over the entire aqueous volume. Each SAR value is a mean of three measurements realized on three different samples.

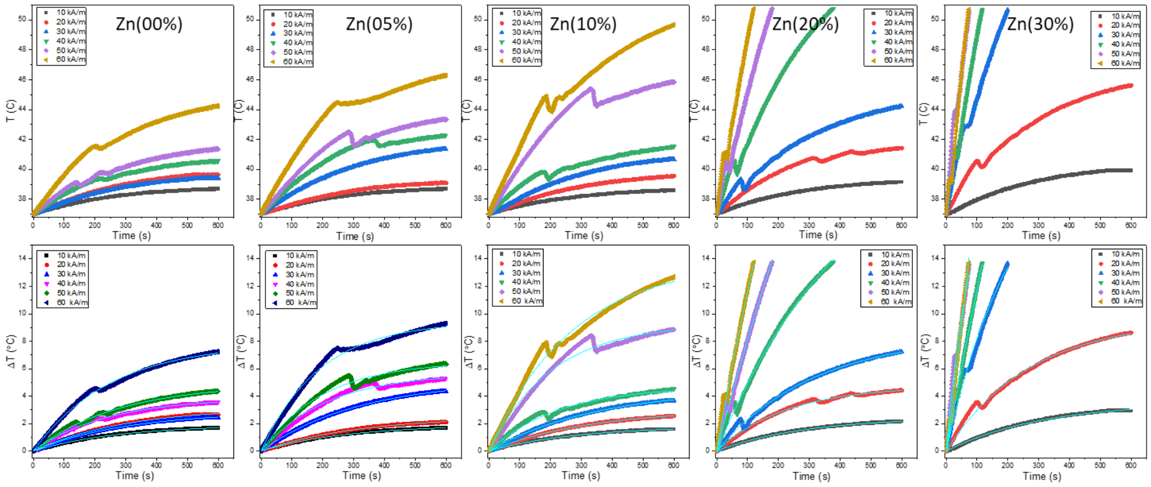

Figure S1. The heating curves (upper panels) and their corresponding temperature change ΔT versus time curves (lower panels) fitted with Box-Lucas equation (blue curves) of Co ferrites particles, dispersed in water at an iron concentration of 1.00 mg_{MNPs}/mL, recorded as a function of H (10 – 60 kA/m, step of 10 kA/m) at frequency of 355 kHz.

References

1. Iacovita, C.; Stiufiuc, R.; Radu, T.; Florea, A.; Stiufiuc, G.; Dutu, A.; Mican, S.; Tetean, R.; Lucaciu, C.M. Polyethylene glycol medi-ated synthesis of cubic iron oxide nanoparticles with high heating power. *Nanoscale Res. Lett.* **2015**, *10*, 1–16. https://doi.org/10.1186/s11671-015-1091-0.
